# Modulation of neural fMRI responses to visual food cues by overeating and fasting interventions: A preliminary study

**DOI:** 10.14814/phy2.14639

**Published:** 2020-12-24

**Authors:** Liya Kerem, Laura Holsen, Pouneh Fazeli, Miriam A. Bredella, Christopher Mancuso, Megi Resulaj, Tara M. Holmes, Anne Klibanski, Elizabeth A. Lawson

**Affiliations:** ^1^ Neuroendocrine Unit Department of Medicine Massachusetts General Hospital Boston MA USA; ^2^ Pediatric Endocrinology Massachusetts General Hospital for Children Boston MA USA; ^3^ Harvard Medical School Boston MA USA; ^4^ Division of Women’s Health Department of Medicine Brigham and Women’s Hospital Boston Ma USA; ^5^ Department of Psychiatry Brigham and Women’s Hospital Boston MA USA; ^6^ Department of Radiology Massachusetts General Hospital Boston MA USA; ^7^ Translational and Clinical Research Center Massachusetts General Hospital Boston MA USA

**Keywords:** diet, eating disorders, fMRI, food, obesity

## Abstract

Neural processing of visual food stimuli is perturbated at extremes of weight. Human fMRI studies investigating diet effects on neural processing of food cues could aid in understanding altered brain activation in conditions of under‐ and overnutrition. In this preliminary study, we examined brain activity changes in response to 10 days of high‐calorie‐diet (HCD), followed by 10 days of fasting, hypothesizing that HCD would decrease activation in homeostatic and reward regions, while fasting would increase activation in homeostatic/reward regions and decrease activation of self‐control regions. Seven adults completed fMRI scanning during a food‐cue paradigm (high‐ and low‐calorie food images and nonfood objects), pre‐ and post‐10‐day HCD. Six adults completed fMRI scanning pre‐ and post‐10‐day fasting. BOLD response changes for contrasts of interest pre‐ versus post‐intervention in regions of interest were examined (peak‐level significance set at p(FWE)<0.05). BMI increased by 6.8% and decreased by 8.1% following HCD and fasting, respectively. Following HCD, BOLD response in the hypothalamus (homeostatic control), was attenuated at trend level in response to high‐ versus low‐calorie foods. Following fasting, BOLD response to food versus objects in inhibitory‐control areas (dorsolateral prefrontal cortex) was reduced, whereas the activation of homeostatic (hypothalamus), gustatory, and reward brain areas (anterior insula and orbitofrontal cortex) increased. Overfeeding and fasting for 10 days modulate brain activity in response to food stimuli, suggesting that in healthy adults, changes in energy balance affect saliency and reward value of food cues. Future studies are required to understand this interaction in states of unhealthy weight.

## 1 INTRODUCTION

The regulation of appetite and food intake is a complex process governed by neural and endocrine signaling in response to energy balance, motivational drive, interoceptive cues, as well as cognitive and attentional processing of external sensory cues. Human neuroimaging studies demonstrate that in healthy individuals, states of hunger and satiety can modulate and bias the neural processing of visual food cues of different caloric value. Using functional magnetic resonance imaging (fMRI) to compare the brain response to high‐ versus low‐calorie visual food images after an overnight fast, healthy participants demonstrated hyperactivation of the hypothalamus, the primary homeostatic control region, as well as brain areas involved in visual processing (inferior temporal visual cortex), attention and motor control (posterior parietal cortex and premotor cortex, and memory (hippocampus) (Cornier et al., 2007). Additionally, activation in reward‐ and interoception‐related brain areas such as the orbitofrontal cortex (OFC) and the insular cortex is increased in response to palatable food images following a short‐term (3 hr) fast (Frank et al., 2010) as well as an overnight fast (Fuhrer et al., 2008; Goldstone et al., 2009) with positive correlations between the duration of caloric deprivation (1 to 16 hr) and degree of neural activation in the OFC, as well as brain areas of attention (anterior cingulate cortex), motivation (putamen), and motor control (precentral gyrus) (Stice et al., 2013).

In comparison, satiation produces a robust decrease in food‐cue elicited brain activity in reward‐related areas (amygdala, parahippocampal gyrus, and anterior fusiform gyrus) (LaBar et al., 2001) as well as increased activation of the dorsolateral prefrontal cortex (DLPFC), an area primarily involved in decision making and exerting inhibitory‐control over hedonic drives (Thomas et al., 2015). Interestingly, when overfed by 30% above caloric need for 2 days and then evaluated after an overnight fast, healthy‐weight individuals demonstrated attenuated hypothalamic activation to high‐calorie visual food images compared with robust activation seen after 2 days of controlled eucaloric diet (Cornier et al., 2007), suggesting that interoceptive signals reflecting physiological needs appropriately bias the cognitive perception of visual food images (Burgess et al., 2018).

While there is ample evidence that healthy‐weight individuals exhibit changes in brain activation in response to visual food images following an overnight fast and postprandially, well‐controlled studies examining fMRI brain activation in response to prolonged (>2 days) fasting and high‐calorie diet interventions resulting in measurable, acute weight changes in humans are lacking. Such studies could improve our understanding of the adaptive neuronal mechanisms in response to more prolonged caloric restriction and overfeeding resulting in weight changes, and thus create the foundations to better understand the neuronal perturbations that occur at extremes of eating behavior, for example, chronic starvation and hyperphagia.

The aim of the present pilot study was to examine changes in fMRI activation in response to 10 days of overfeeding intended to result in 7%–10% weight gain, followed by 10 days of complete caloric deprivation, separated by a 2‐week interval of unsupervised diet. We hypothesized that in healthy men and women, excessive caloric consumption leading to acute weight gain would result in diminished fMRI activation of homeostatic and reward regions important for food processing specifically in response to high‐calorie food images, whereas complete caloric deprivation resulting in acute weight loss would lead to increased activation of homeostasis and reward areas and decreased activation of inhibitory control brain regions in response to images of food varying in caloric content.

## 2 METHODS

### 2.1 Subjects

Healthy, adult participants were recruited as part of a multidisciplinary study designed to examine the effects of acute weight changes on skeletal and marrow adipose tissue remodeling. All subjects included in the current fMRI study performed both diet interventions—overfeeding and fasting, in the same order (for details, see Procedure). Seven subjects (2 men, 5 women; mean age ± *SEM*: 31 ± 2.9 years; mean BMI ± *SEM*: 26.4 ± 0.5 kg/m^2^) completed fMRI scanning pre‐ and post‐10‐day HCD. Six subjects (1 man; 5 women; mean age: 29 ± 1.9 years; mean BMI: 26.6 ± 0.6 kg/m^2^) completed fMRI scanning pre‐ and post‐10‐day fast. Overall, three participants performed all four scans (prior and following each diet intervention). Subjects were recruited through online advertisements. Inclusion criteria included 101%–120% of ideal body weight (to avoid a potential negative impact due to the fasting intervention) and regular menses for women (cycling every 21–35 days). Exclusion criteria included a history of chronic illness, untreated thyroid dysfunction, history of an eating disorder, active substance abuse, abnormal transaminase levels (AST/ALT), hypokalemia (potassium < 3.5 mEq/L), pregnancy, breastfeeding, and MRI contraindications. Due to the main study aim to investigate bone health, participants taking medications known to affect bone metabolism (e.g., systemic steroids and immunosuppressants) were excluded from the study.

### 2.2 Procedure

This study was approved by the Partners HealthCare Institutional Review Board and conducted in accordance with the Declaration of Helsinki and was preregistered at clinicaltrials.gov (NCT02482519). All subjects provided written informed consent prior to participation, obtained by a licensed physician investigator or nurse practitioner. Subjects were admitted to the Translational and Clinical Research Center (TCRC) at Massachusetts General Hospital (MGH) for an outpatient screening visit including a medical history, physical exam, and blood work to determine eligibility. Eligible subjects were admitted to the TCRC for the main study visits which occurred over the course of 35 days. The main study included an initial 10‐day inpatient HCD weight‐gain period (days 1–11), followed by a 13–18 day outpatient unsupervised diet (“wash‐out,” days 11–24), followed by a 10‐day inpatient fast (days 25–35). During the HCD intervention, subjects were fed a high fat/high carbohydrate diet individually tailored to result in a 7%–10% weight gain, planned and supervised by an experienced bionutritionist at the TCRC. The amount of daily calories ordered for each participant was established by calculating the Basal Metabolic Rate (using the Mifflin St Jeor equation) and adding 20% on top of the final result to achieve a positive energy balance (Frankenfield et al., 2005). During the overfeeding intervention, the study nutritionist examined the daily percent change in body weight and evaluated meal completion to assess the need for diet modifications. When participants were able to fully complete their meal but did not gain enough weight, food items were altered to increase the daily caloric consumption. When participants were struggling to complete their meal, a high‐calorie milkshake was added in addition to their meal. After a wash‐out period, subjects were readmitted to the TCRC for a 10‐day fast, using a validated protocol (Fazeli et al., 2015). During fasting, subjects were given water ad libitum, 20 meq of oral potassium chloride daily to prevent hypokalemia, and a multivitamin. During the inpatient portions of the study, subjects were free to move around the TCRC or remain in their room where they had a TV. Participants remained inpatient for 24hr following the fasting intervention to gradually increase caloric intake under medical supervision. All subjects were blinded to their weights during the entire study.

### 2.3 Functional MRI Paradigm

Brain fMRI was performed after an overnight fast on the first and last day of both diet interventions. A urine HCG test was completed in females prior to scanning. fMRI scanning was performed during a well‐established food motivation paradigm (Holsen et al., 2012) programmed through Presentation software (Neurobehavioral Systems). Briefly, at each visit, subjects viewed 100 high‐calorie food stimuli, 100 low‐calorie food stimuli, 100 non‐food‐related household objects, and 100 fixation stimuli (blurred images) in a block design, with each stimulus presented for 3 s. Subjects were instructed to press a button when pictures changed to ensure attention. A total of five 4‐min runs with five images in each block and 16 blocks in each run were completed at each visit. At each of the pre and postintervention visits, subjects viewed images from one of two parallel versions of the task (each with 400 unique images from the conditions above), with order randomized across subjects.

### 2.4 MRI Acquisition Parameters

MRI data were acquired using a Siemens 3T Trio scanner (Siemens) at the Athinoula A. Martinos Center for Biomedical Imaging. A gradient‐echo EPI pulse sequence was used (33 contiguous oblique‐axial slices, 4‐mm thick, TR/TE = 2000/30 ms, flip angle = 90°, FOV = 200 × 200 mm, 120 total images per run). A sagittal 3D SPGR (T1‐weighted) sequence was acquired (TR/TE = 2350/3.39 ms, flip angle = 7°, FOV = 256 × 256 mm, effective slice thickness = 1.33 mm with 128 slices) for co‐registration between structural and functional datasets. Head movements were restricted with foam cushions.

### 2.5 Data analysis

fMRI data were analyzed using Statistical Parametric Mapping software (SPM8; Wellcome Trust Centre for Neuroimaging). Volumes were realigned and unwarped with phase correction provided from the fieldmap, normalized to the Montreal Neurological Institute MNI152 brain template, re‐sampled to 3 mm isotropic, and smoothed with a 6 mm Gaussian kernel. Outliers in the global mean image time series (threshold: 3.5 *SD*) and movement (threshold: 0.8 mm, scan‐to‐scan movement) were detected using ART (http://www.nitrc.org/projects/artifact_detect/) and entered as nuisance regressors in the single‐subject level GLM. Masks excluding voxels outside the brain were applied to ensure that voxels in regions with signal dropout were not arbitrarily excluded. For the block design, each stimulus type was modeled using a boxcar function convolved with a canonical hemodynamic response function. Contrasts of interest (food > objects; high‐calorie food > low‐calorie food) from the first‐level analysis were tested using linear contrasts and SPM t‐maps, then submitted to second‐level random‐effects group analysis. The contrast of high‐calorie food > low‐calorie food was chosen as the contrast of interest for the pre‐ versus post‐HCD analysis based on the prediction that this contrast would be uniquely sensitive to changes induced by the high‐calorie diet foods consumed during the HCD intervention. Conversely, the food > objects contrast was selected for the pre‐ versus post‐fasting analysis based on the expectation that this more general contrast would be specifically sensitive to changes induced by lack of intake of any food during the fasting intervention. Effects of interest at the group level were examined using paired *t*‐tests (pre‐ vs. post‐HCD; pre‐ vs. post‐fast). For transparency, post hoc complementary analyses were completed to examine changes in response to food > objects contrast for the pre‐ to post‐HCD intervention, and changes in response to high‐calorie food > low‐calorie food contrast for the pre‐ to post‐fasting intervention. For each set of analyses, region of interest (ROI) analyses were performed using small volume correction which corrects for the number of voxels within an anatomical ROI. Multiple comparisons were controlled using a combination of cluster extent (k ≥ 5 in hypothalamus and nucleus accumbens; k ≥ 20 in other ROIs) and *p* < .05 FWE‐corrected thresholds. ROI were selected a priori and included the hypothalamus, nucleus accumbens, OFC, amygdala, insula, and dorsolateral prefrontal cortex (DLPFC). Anatomical borders of the hypothalamus, nucleus accumbens, OFC, amygdala, and insula were defined using a manually segmented MNI brain [based on methods established by the Center for Morphometric Analysis at Massachusetts General Hospital and Harvard Medical School (Makris et al., 2006, 2013). Anatomical borders of the DLPFC were derived from Middle Frontal Gyrus region as defined by the AAL atlas (Tzourio‐Mazoyer et al., 2002). Average parameter estimates within each ROI for each participant were extracted from clusters within each ROI meeting statistical thresholds using the Region of Interest Extraction Toolbox (REX) and exported to SPSS (v19, Chicago, IL) for graphical depiction.

## 3 RESULTS

**FIGURE 1:**
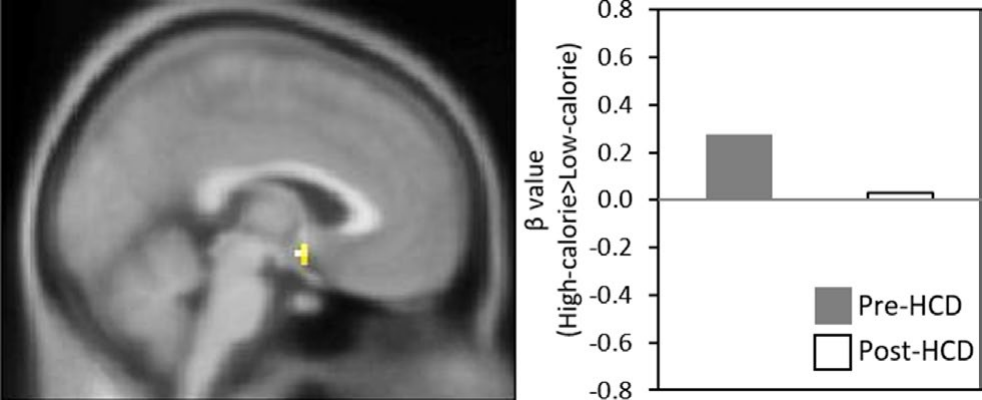
Change in Neural Response to High‐ versus Low‐calorie Food before and after HCD. Figure shows SPM map of BOLD activation at *p*_FWE_ = 0.069 in the right hypothalamus in response to High‐calorie > Low‐calorie food from Pre‐HCD > Post‐HCD, overlaid on the avg152T1 template. Bar graph on the right of the figure visually presents the mean BOLD response to the High‐calorie > Low‐calorie food contrast within the cluster (*k* = 5) meeting statistical thresholds (defined in the Methods; see Table 1 for MNI coordinates) for the Pre‐HCD (gray bar) versus Post‐HCD (white bar) comparison

Mean BMI increased by 6.8%±0.7 following HCD, was almost fully restored at the end of the wash‐out period and decreased by 8.1%±0.6 following the fasting intervention (see Table 1). Following HCD intervention, hypothalamic BOLD response was attenuated at a trend level in response to high‐ versus low‐calorie food images (Table 2; Figure 1). There were no regions in which BOLD response to all food images *(high‐calorie + low‐calorie)* versus objects changed significantly following HCD intervention.

**TABLE 1:** Means and ranges of BMIs before and after each diet intervention

BMI	HCD (*n* = 7)		Fasting (*n* = 6)	
	Pre‐HCD	Post‐HCD	Pre‐fasting	Post‐fasting
Mean	26.4 kg/m^2^	28.1 kg/m^2^	26.6 kg/m^2^	24.5 kg/m^2^
Range	23.7–27.9 kg/m^2^	24.8–30.0 kg/m^2^	24.0–27.9 kg/m^2^	22.0–26.3 kg/m^2^

Abbreviation: HCD, High‐calorie diet.

**TABLE 2:** Change in BOLD response to food stimuli following 10‐day HCD and 10‐day fast

Condition	ROI	Hemisphere	*k* (E)	*x*	*y*	*z* ^a^	*t*	Uncorrected *p*‐value^b^	Peak‐level *P_FWE‐corr_* ^c^	Cohen's *d’*
Pre‐HCD > Post‐HCD: High‐calorie > Low‐calorie										
	Hypothalamus	R	5	0	2	−8	3.83	.004	0.069	0.39
Pre‐Fast > Post‐Fast: Food > Objects										
	DLPFC	L	191	−36	20	37	12.27	<.001	**0.049**	**2.07**
Post‐Fast > Pre‐Fast: Food > Objects										
	Hypothalamus	R	21	6	−1	−17	6.64	.001	**0.050**	**2.65**
	OFC	R	361	30	20	−26	12.81	<.001	**0.036**	**5.99**
	Insula	L	55	−39	5	−14	13.86	<.001	**0.014**	**4.87**

^a^ Coordinates are presented in MNI space.

^b^ Voxel‐wise Z‐score significance level *p* < .05 uncorrected for multiple comparisons within a hypothesized ROI; ROIs listed represent regions of significantly activated clusters within the a priori hypothesized ROI.

^c^ FWE rate (family‐wise error rate) used for SVC (small volume correction): Peak‐level significance level (FWE‐corrected within the search volume of interest); p values for ROIs reaching p(FWE‐corrected)<0.05 are **bolded.**

**FIGURE 2:**
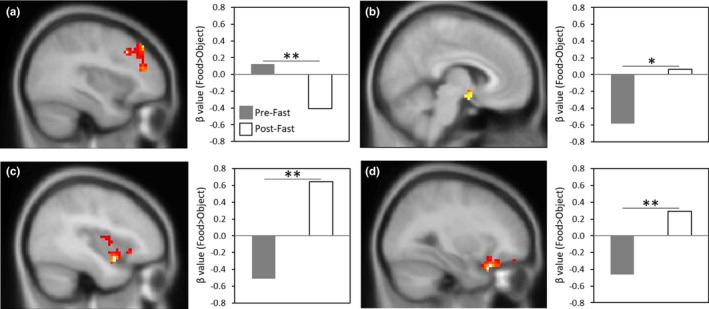
Change in Neural Response to Food versus Objects before and after Fasting. For each ROI, figures show SPM maps of BOLD activation at *p*_FWE_ < 0.05 in response to Food > Objects from Pre‐ to Post‐Fast, overlaid on the avg152T1 template. Bar graphs on the right of each figure visually present the mean BOLD response to the Food > Object contrast within clusters meeting statistical thresholds (defined in the Methods; see Table 2 for MNI coordinates) for the Pre‐Fast (gray bar) versus Post‐Fast (white bar) comparison: (a) left DLPFC; (b) right hypothalamus; (c) left insula; (d) right OFC. *p = .05, FWE‐corrected; **p < .05, FWE‐corrected

Following the 10‐day fast, BOLD response in the DLPFC was reduced in response to food images compared with objects (Table 2; Figure 2a). Conversely, following the fasting intervention, BOLD response to food versus objects increased significantly in the OFC and insula and at a trend level in the hypothalamus (Table 2; Figure 2b‐d). The 10‐day fast did not induce significant BOLD changes in a priori ROIs in response to high‐ versus low‐calorie food images.

## 4 DISCUSSION

In this preliminary, proof‐of‐concept study, we showed for the first time that when healthy adults were exposed to 10 days of extreme diet intervention associated with acute weight changes, they demonstrated altered neural processing of visual food images in brain regions involved in homeostatic control, reward, interoception, and inhibitory control. Specifically, following an overfeeding intervention associated with a mean BMI increase of 6.8%, subjects exhibited trend‐level attenuated hypothalamic activation, reflecting homeostatic control, in response to high‐ versus low‐calorie foods. Conversely, 10 days of fasting induced a mean BMI decrease of 8.1%, reduced activation in response to food images versus objects in inhibitory control areas (DLPFC), and increased activation in homeostatic (hypothalamus), interoception (anterior insula), and reward valuation (orbitofrontal cortex) brain regions. There is strong evidence that in healthy individuals, fMRI activation in food motivation brain areas is altered in states of short‐term hunger and satiety (Cornier et al., 2007; Frank et al., 2010; Goldstone et al., 2009; LaBar et al., 2001). However, the majority of these studies examined the effects of an overnight fast or a meal on the brain response to food cues and were not associated with a change in weight status. Our novel study design allowed an evaluation of the neurobiological response to visual food images following a relatively prolonged diet intervention resulting in significant acute weight changes, thus more closely mimicking extremes of disordered eating leading to unhealthy weight and associated co‐morbidities.

In our study, following 10 days of overfeeding, subjects demonstrated trend‐level attenuated hypothalamic activation in response to high‐calorie versus low‐calorie food images. The hypothalamus is critically involved in integrating internal homeostatic signals with external cues and input from high‐order brain regions important for emotional, cognitive, and reward processing and it has been implicated in the pathogenesis of obesity (Roth et al., 2019; Thaler et al., 2012). More specifically, rodent studies show that caloric excess triggers an inflammatory hypothalamic response altering the intricate neuroendocrine hypothalamic network regulating appetite and energy balance (Razolli et al., 2019). Hypothalamic inflammation is associated with the proliferation of glial cells which can be detected in human structural MRI studies. Signs of hypothalamic gliosis and inflammation can be seen in adults and even children with obesity and are positively associated with adiposity (Schur et al., 2015; Sewaybricker et al., 2019). fMRI studies provide further insight into the hypothalamic perturbations associated with obesity. While healthy‐weight individuals demonstrate dose‐dependent reductions in hypothalamic fMRI signal following glucose ingestion (Smeets et al., 2005) and in response to high‐calorie food images following overfeeding for 2 days (Cornier et al., 2009), individuals with overweight/obesity do not show the same hypothalamic response (Cornier et al., 2009; Matsuda et al., 1999). Our finding of trend‐level attenuated hypothalamic activation in response to high‐ versus low‐calorie food images following 10 days of caloric excess, suggests that differential hypothalamic responsivity to external food cues representing divergent rewarding value is maintained following 10 days of overfeeding associated with weight gain. These findings are of interest since rodent studies show that 3 days of high‐fat diet are sufficient to induce proinflammatory hypothalamic response associated with altered mitochondrial function and synaptic plasticity as well as impaired functional and molecular activation of insulin‐signaling pathways (Jais & Bruning, 2017; McLean et al., 2019; De Souza et al., 2005).

The prolonged fasting intervention implemented in our study provides a model through which to understand restrictive eating disorders such as anorexia nervosa (AN), a condition associated with severe, chronic starvation. AN is associated with dysfunction of appetite‐regulating brain areas (Lloyd & Steinglass, 2018), including hypoactivation of these areas (e.g., insula, OFC, and hypothalamus) in response to high‐calorie foods after a 12‐hr overnight fast (Holsen et al., 2012) and hyperactivation of the DLPFC in response to high‐calorie food images (Brooks et al., 2011). Altered reward and interoceptive processing of hedonic food cues (e.g., palatable food images and tastes of sucrose) as seen in fMRI studies and following a 12–16 hr fast also exists in women who remitted from AN, thus eliminating a confounding factor of malnutrition on food motivation neurocircuitry response and supporting pathological inherent mechanisms facilitating extended periods of food avoidance (Holsen et al., 2012; Kaye et al., 2020). Our subjects demonstrated clear hyperactivation of homeostatic and food motivation brain areas as well as hypoactivation of the DLPFC following 10‐day fasting, which we speculate could provide a neural signature of the state of acute starvation in otherwise healthy adults. Since most fMRI studies designed to learn about differences in food‐cue processing between AN patients and healthy controls were conducted following a short‐term fast, our study adds to the literature by providing greater resemblance to the prolonged starvation associated with weight loss seen in AN. Additional studies are needed to further delineate the underlying neurobiological mechanisms of chronic starvation observed in AN. Our study design may also provide important information regarding neurobiological features of food processing in atypical anorexia nervosa (AAN), a relatively new diagnosis introduced in the DSM‐5 (APA, 2013). Patients with AAN have similar features to patients with AN (e.g., significant food restriction and intense fear of weight gain), however, they are not underweight; rather, they have BMIs in the normal to overweight/obesity range (Matthews et al., 2019; Moskowitz & Weiselberg, 2017). fMRI studies examining food cues processing in patients with AAN are lacking. Future studies comparing fMRI responses to food cues in patients with AAN compared to BMI‐matched controls could provide important knowledge regarding perturbation in the neurocircuitry of eating behavior in AAN. Finally, it is also important to note that prolonged fasting (greater than ~14 hr), is associated with significant cellular, metabolic and endocrine adaptions that could potentially affect appetite and subsequently fMRI activation in response to visual food stimuli. Prolonged fasting is associated with a “metabolic switch,” transitioning from using glucose and carbohydrates as fuel sources for both body and brain to fatty acids and ketones (Mattson et al., 2017). It is also associated with marked changes in appetite‐regulating hormones such as leptin, insulin, ghrelin, and cortisol (Freire & Alvarez‐Leite, 2020; Rajpal & Ismail‐Beigi, 2020; Steinhauser et al., 2018). Future studies comprehensively evaluating both neural activation to food stimuli together with neuroendocrine adaptation to prolonged fasting will be important to conduct.

Our findings are limited by the small sample size, although the significant effects observed following the diet interventions suggest adequate power for the statistical analysis approach implemented in this pilot study. Additionally, to avoid the potential harmful consequences of the fasting intervention, participants recruited to this study were on average slightly overweight (101%–120% of ideal body weight). Since chronic obesity is associated with altered fMRI brain activation in response to high‐calorie food images (Devoto et al., 2018), the mild excess weight of the participants could have affected the results seen in our investigation. Another limitation relates to the known gender differences in visual processing of food images in hunger and satiety (Chao et al., 2017) that we could not capture in our small sample. It is also important to note that three of the total number of subjects who participated in this study performed both fMRI evaluations (pre‐ and post‐overfeeding and fasting), and we cannot exclude a confounding effect of the familiarity with the visual stimuli presented during the fMRI task. Finally, the brain activation observed following fasting in this study could have been impacted by the prior high‐calorie diet intervention which all subjects received due to the study design. Future similar studies could include a randomized, crossover study design to minimize the potential confounding effect of participating in the two diet interventions in the same order (overfeeding followed by fasting).

In summary, our preliminary, novel findings demonstrate that 10 days of extreme diet intervention modulate brain activation in regions related to homeostasis, reward, interoception, and inhibitory control in a nutritional status‐dependent manner. A comprehensive and mechanistic understanding of the neurophysiology underlying states of hunger and satiety in healthy individuals could support research of disordered eating.

## CONFLICT OF INTEREST

None.

## DISCLOSURE

Dr. Lawson has served on the scientific advisory board and has a financial interest in OXT Therapeutics, a company developing an intranasal oxytocin and long‐acting analogs of oxytocin to treat obesity and metabolic disease. Her interests were reviewed and are managed by Massachusetts General Hospital and Partners HealthCare in accordance with their conflict of interest policies. This company was not involved in any way in this research. Dr. Klibanski reports that she is a consultant for Chiasma, on the Scientific Advisory Board for Crinetics, and is the recipient of an investigator‐initiated grant to MGH from Ipsen. The other authors have no conflict of interest.

## AUTHORS’ CONTRIBUTIONS

LK, LH, PF, AK, and EAL were involved in the conception and design of the study. LK, LH, PF, AK, and EAL carried out the interpretation of experimental results. LK and LH performed data analysis and figure preparation. CM, MR, and TMH were involved in the conduction of experiments. LK, LH, and EAL carried out the drafting of the manuscript. All authors revised and approved the final version of the manuscript.
